# Valency-based structural properties of gamma-sheet of boron clusters

**DOI:** 10.1371/journal.pone.0303570

**Published:** 2024-05-23

**Authors:** Ali N. A. Koam, Muhammad Azeem, Ali Ahmad

**Affiliations:** 1 Department of Mathematics, College of Science, Jazan University, New Campus, Jazan, Saudi Arabia; 2 Department of Mathematics, Riphah International University, Lahore, Pakistan; 3 College of Computer Science & Information Technology, Jazan University, Jazan, Saudi Arabia; Guizhou University of Finance and Economics, CHINA

## Abstract

Boron cluster sheets are two-dimensional boron atom-based formations called borophene. They are similar to the two-dimensional sheet known as graphene, which is composed of carbon atoms arranged in a hexagonal lattice. The unique electrical, mechanical, and thermal properties of borophene make it a sought-after substance for a variety of uses, such as catalysis, energy storage, and electronics. There are two ways to manufacture borophene: chemical vapor deposition and molecular beam epitaxy. Vertex-edge valency-based topological descriptors are a great example of a molecular descriptor that provides information on the connection of atoms in a molecule. These descriptions are based on the notion that a node’s value in a molecular network is the sum of the valency of those atoms that are directly connected to that node. In this article, we discussed some novel vertex-edge (*ve*) and edge-vertex (*ev*) topological descriptors and found their formulations for the boron cluster or borophene sheets. Also, we show the numerical and graphical comparison of these descriptors in this article.

## 1 Introduction

A fundamental study in the structural theory of chemical graphs is a molecular structure diagram, in which atoms are seen as atoms and lines as chemical bonds. If there is a path connecting any two atoms in a diagram, it is said to be linked. A system is a linked map with a loop and no other lines separating any two atoms. The number of atoms is linked to a given node. The valency of *v* is indicated by the symbol *d*_*v*_. The open neighbourhood of node *v* is the collection of all the atoms that are close to it, and it may be represented by the symbol *N*(*v*). When the node *v*, indicated by *N*[*v*], was included, the open neighbourhood changed to the closed neighbourhood.

The total length of the most constrained path between two atoms is the distance across both. In physical science, the concepts of valence and valency are pretty closely related. For basic diagram invariants, see [1]. The qualities and unstudied material’s natural exercises are predicted by the relationship between the QSPR and QSAR. Topological descriptors and a few physicochemical features are used in such compounds for estimating the bioactivity of chemical substances [2–5]. In a diagram of a chemical compound, a number is used to express a topological descriptor that may be used to depict the highlighted chemical molecule and predict its physiochemical characteristics.

Wiener devised the topological descriptor structure in 1947. He presented the Wiener descriptor and was given an approximation of the alkanes’ point of boiling [6–10]. More than 3000 topological indicators have been characterized so far, however, no one descriptor is enough to determine all physicochemical characteristics, yet these topological descriptors together can partly do this. After that the Randić descriptor was given by cite7 in 1975. One of the most significant, widely used, and applicable topological descriptors is the Randić descriptor. Graph invariance is the subject of several surveys, studies, and books [11–16]. For further detail, we refer to see [17–22].

Authors of [23], first proposed two unique valency concepts, the *ve*-valency and the *ev*-valency, and [24, 25] made a contribution to the research on the “*ve*-valency” and the “*ev*-valency”. When current descriptors were combined with the newly developed valency-based descriptors, the outcomes were improved, as shown in [26–28]. It is currently shown that the *ve*-valency Zagreb descriptor has a stronger approximation performance than the original Zagreb descriptor.

There are maany more graph theoretical parameters other than topological descriptors like resolvability parameters [29–42].

## 2 The *ve*-valency and *ev*-valency based topological descriptors

The definition of an edge’s *ev*-valency was provided by the contributors of citation number [23], and it is the count of atoms in the union of the closed neighbourhoods of *u* and *v*, where *e* = *uv* ∈ *E* which is denoted by *d*_*ev*_(*e*). The count of routes of various lines that are incident to any node from the closed neighbourhood of *v* is known as the *ve*-valency of the node *v* ∈ *V*, indicated by the symbol *d*_*ve*_(*v*). For the sake of this essay, we will assume that *G* is a connected graph with *e* = *uv* ∈ *E*(*G*) while *v* ∈ *V*. In this section, we provide some fundamental notions of *ve*-valency and *ev*-valency topological descriptors, [23, 43]. Some *ev*-valency related topological descriptors are: the *ev*-valency Randić descriptor, ansd *ev*-valency Zagreb descriptor, and topological descriptors related to *ev*-valency are: The first *ve*-valency Zagreb *α* descriptor, *ve*-valency sum-connectivity descriptor, *ve*-valency harmonic descriptor, *ve*-valency geometric-arithmetic descriptor, *ve*-valency atom-bond connectivity descriptor, *ve*-valency Randić descriptor, second *ve*-valency Zagreb descriptor, and first *ve*-valency Zagreb *β* descriptor. All these descriptors for any graph *G* can be found in [44], are formulated as:

- The *ev*-valency Zagreb Index: $M^{ev}\left(G\right)=\sum_{e\in E(G)}d_{ev}{\left(e\right)}^{2}$
- The first *ve*-valency Zagreb *α* descriptor: $M_{1}^{\alpha ve}\left(G\right)=\sum_{v\in V(G)}d_{ve}{\left(v\right)}^{2}$
- The first *ve*-valency Zagreb *β* descriptor: $M_{1}^{\beta ve}\left(G\right)=\sum_{uv\in E(G)}\left(d_{ve}\left(u\right)+d_{ve}\left(v\right)\right)$
- The second *ve*-valency Zagreb descriptor: $M_{2}^{ve}\left(G\right)=\sum_{uv\in E(G}\left(d_{ve}\left(u\right)\times d_{ve}\left(v\right)\right)$
- The *ve*-valency Randić descriptor: $R^{ve}\left(G\right)=\sum_{uv\in E(G}{\left(d_{ve}\left(u\right)\times d_{ve}\left(v\right)\right)}^{-\frac{1}{2}}$
- The *ev*-valency Randić descriptor: $R^{ev}\left(G\right)=\sum_{e\in E(G)}d_{ev}{\left(e\right)}^{-\frac{1}{2}}$
- The atom-bond connectivity descriptor: ${ABC}^{ve}\left(G\right)=\sum_{uv\in E(G)}\sqrt{\frac{d_{ve}\left(u\right)+d_{ve}\left(v\right)-2}{d_{ve}\left(u\right)\times d_{ve}\left(v\right)}}$
- The geometric-arithmetic descriptor: ${GA}^{ve}\left(G\right)=\sum_{uv\in E(G)}\frac{2\sqrt{deg_{ve}\left(u\right)\times d_{ve}\left(v\right)}}{d_{ve}\left(u\right)+d_{ve}\left(v\right)}$
- The harmonic descriptor: $H^{ve}\left(G\right)=\sum_{uv\in E(G)}\frac{2}{d_{ve}\left(u\right)+d_{ve}\left(v\right)}$
- The sum-connectivity descriptor: $\chi^{ve}\left(G\right)=\sum_{uv\in E(G)}{\left(d_{ve}\left(u\right)+d_{ve}\left(v\right)\right)}^{-\frac{1}{2}}$

## 3 The *γ*−sheet of boron clusters

**Fig 1:**
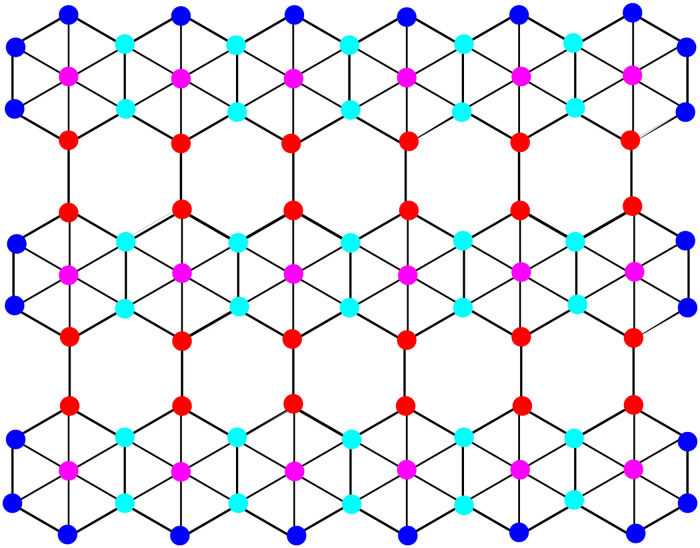
*γ*−sheet of Boron Clusters for *p* = 6 and *q* = 3.

Boron cluster sheets are two-dimensional boron-atom-based formations called borophene. They are similar to the two-dimensional sheet known as graphene, which is composed of carbon atoms arranged in a hexagonal lattice. The unique electrical, mechanical, and thermal properties of borophene make it a sought-after substance for a variety of uses, such as catalysis, energy storage, and electronics. There are two ways to manufacture borophene: chemical vapour deposition and molecular beam epitaxy. To create borophene sheets, many arrangements of boron atoms, such as triangular, honeycomb, and rectangular patterns, may be created. Fig 1 shows the a graph of *γ*−sheet of boron clusters for the particular values of *p* = 6 and *q* = 3. We established our computational findings in this part for *γ*−sheet of boron clusters (it is denoted by *γ*(*p*, *q*)). The count of atoms and edges in *γ*(*p*, *q*) are 5*pq* + 2*q* and 12*pq* − *p* + *q* respectively, shown in the Table 1.

**Table 1:** Numerical comparison of $M^{ev},M_{1}^{\alpha ve},M_{1}^{\beta ve}$, $M_{2}^{ve}$ and *R*^*ev*^.

Notations	Detail
*G*	A simple connected graph
*d*(*v*)	Degree (valency) of the vertex *v*
*d*_*ev*_(*v*)	*ev* degree of the vertex *v*
*d*_*ve*_(*v*)	*ve* degree of the vertex *v*

In Table 2, we partitioned the lines based on *ev*-valency of the *γ*(*p*, *q*). In Table 3, we partition the atoms based on *ve*-valency of *γ*(*p*, *q*). We partition the lines based on *ve*-valency of the end atoms of *γ*(*p*, *q*) in Table 4.

**Table 2:** Line partition of *γ*(*p*, *q*).

Number of lines	Degree of its end atoms	*ev*-valencys
2*q* + 4	(3, 3)	6
4*q* − 4	(3, 4)	7
4*p* − 4	(3, 5)	8
2*p* + 4*q*	(3, 6)	9
*p*(*q* − 1)	(4, 4)	8
4(*p* − 1)(*q* − 1)	(4, 5)	9
2*p*(*q* − 1)	(4, 6)	10
(*p* − 1)*q*	(5, 5)	10
4(*p* − 1)*q*	(6, 6)	12

**Table 3:** Node partition of *γ*(*p*, *q*).

Degree of atoms	*ve*-valencys	Number of atoms
3	12	4
3	13	4*q* − 4
3	14	4
3	16	2*p* − 4
4	18	4*q* − 4
4	20	2(*p* − 2)(*q* − 1)
5	23	2(*p* − 1)
5	25	2(*p* − 1)(*q* − 1)
6	23	4
6	24	2*q* − 4
6	27	2*p* − 4
6	28	(*p* − 2)(*q* − 2)

**Table 4:** The line partition of *ve*-valency based of its end atoms of *γ*(*p*, *q*).

vertex-edge based partition					
(*d*_*u*_, *d*_*v*_)	(*S*_*u*_, *S*_*v*_)	Frequency	(*d*_*u*_, *d*_*v*_)	(*S*_*u*_, *S*_*v*_)	Frequency
(3, 3)	(12, 13)	4	(4, 5)	(18,25)	4*q* − 4
(3, 3)	(12, 14)	4	(4, 5)	(20,25)	4(*p* − 2)(*q* − 1)
(3, 3)	(13, 13)	2*q* − 4	(4, 6)	(18,23)	4
(3, 4)	(13, 18)	4*q* − 4	(4, 6)	(18,24)	4(*q* − 2)
(3, 5)	(14, 23)	4	(4, 6)	(20,27)	2(*p* − 2)
(3, 5)	(16, 23)	4*p* − 8	(4, 6)	(20,28)	2(*p* − 2)(*q* − 2)
(3, 6)	(12, 23)	4	(5, 5)	(23,25)	2(*p* − 1)
(3, 6)	(13, 23)	4	(5, 5)	(25,25)	(*p* − 1)(*q* − 2)
(3, 6)	(13, 24)	4*q* − 8	(5, 6)	(23,23)	4
(3, 6)	(14, 23)	4	(5, 6)	(23,25)	4
(3, 6)	(16, 27)	2*p* − 4	(5, 6)	(23,27)	4(*p* − 2)
(4, 4)	(18, 18)	2*q* − 2	(5, 6)	(25,27)	4*p* − 8
(4, 4)	(20, 20)	(*p* − 2)(*q* − 1)	(5, 6)	(24,25)	4(*q* − 2)
			(5, 6)	(25,28)	4(*p* − 2)(*q* − 2)

## 4 Main results

In this part, we will determine different kinds of descriptors’ *ev*-valency and *ve*-valency based descriptors, which are listed as follows;

- ***ev*−valency Zagreb Index**.

Using the statistics given in Table 2, we determine the *ev*-valency based Zagreb descriptor of *γ*(*p*, *q*) as:

$$\begin{array}{ccc} M^{ev}\left(\gamma \left(p,q\right)\right) & = & \sum_{e\in E(\gamma (p,q))}d_{ev}{\left(e\right)}^{2},\\ M^{ev}\left(\gamma \left(p,q\right)\right) & = & \left(2q+4\right)\times 6^{2}+\left(4q-4\right)\times 7^{2}+\left(4p-4\right)\times 8^{2}+\left(2p+4q\right)\times 9^{2}\\ & + & p\left(q-1\right)\times 8^{2}+4\left(p-1\right)\left(q-1\right)\times 9^{2}+2p\left(q-1\right)\times 10^{2}\\ & + & \left(p-1\right)q\times 10^{2}+4\left(p-1\right)q\times 12^{2}\\ & = & 1264pq-170p-408q+16. \end{array}$$

- **The first *ve*-valency Zagreb *α* descriptor.**

Using the statistics given in Table 3, we determine the *ve*-valency based Zagreb descriptor of *γ*(*p*, *q*) as:

$$\begin{array}{ccc} M_{1}^{\alpha ve}\left(\gamma \left(p,q\right)\right) & = & \sum_{v\in V(\gamma (p,q))}d_{ve}{\left(v\right)}^{2},\\ M_{1}^{\alpha ve}\left(\gamma \left(p,q\right)\right) & = & 4\times 12^{2}+\left(4q-4\right)\times 13^{2}+4\times 14^{2}+\left(2p-4\right)\times 16^{2}\\ & + & \left(4q-4\right)\times 18^{2}+2\left(p-2\right)\left(q-1\right)\times 20^{2}+2\left(p-1\right)\times 23^{2}+2\left(p-1\right)\left(q-1\right)\times 25^{2}\\ & + & 4\times 23^{2}+\left(2q-4\right)\times 24^{2}+\left(2p-4\right)\times 27^{2}+\left(p-2\right)\left(q-2\right)\times 28^{2}\\ & = & 2834pq-590p-1294q+188. \end{array}$$

- **The first *ve*-valency Zagreb *β* descriptor**.

Using the statistics given in Table 4, we determine the *ve*-valency based Zagreb descriptor of *γ*(*p*, *q*) as:

$$\begin{array}{ccc} M_{1}^{\beta ve}\left(\gamma \left(p,q\right)\right) & = & \sum_{uv\in E(\gamma (p,q))}\left(d_{ve}\left(u\right)+d_{ve}\left(v\right)\right),\\ M_{1}^{\beta ve}\left(\gamma \left(p,q\right)\right) & = & 4\times 25+4\times 26+(2q-4)\times 26+(4q-4)\times 31+4\times 37+(4p-8)\times 39\\ & + & 4\times 35+4\times 36+(4q-8)\times 37+4\times 37+(2p-4)\times 43\\ & + & (2q-2)\times 36+(p-2)(q-1)\times 40+(4q-4)\times 43+4(p-2)(q-1)\times 45\\ & + & 4\times 41+4(q-2)\times 42+2(p-2)\times 47+2(p-2)(q-2)\times 48\\ & + & 2(p-1)\times 48+(p-1)(q-2)\times 50+4\times 46+4\times 48+4(p-2)\times 50\\ & + & 4(q-2)\times 49+(4p-8)\times 52+4(p-2)(q-2)\times 53\\ & = & 578pq-96p-174q+16. \end{array}$$

- **The second ve-valency Zagreb descriptor**.

Using the statistics given in Table 4, we determine the *ve*-valency based Zagreb descriptor of *γ*(*p*, *q*) as:

$$\begin{array}{ccc} M_{2}^{ve}\left(\gamma \left(p,q\right)\right) & = & \sum_{uv\in E(\gamma (p,q)}\left(d_{ve}\left(u\right)\times d_{ve}\left(v\right)\right),\\ M_{2}^{ve}\left(\gamma \left(p,q\right)\right) & = & 4\times 156+4\times 168+(2q-4)\times 169+(4q-4)\times 234+4\times 322+(4p-8)\times 368\\ & + & 4\times 276+4\times 299+(4q-8)\times 312+4\times 322+(2p-4)\times 432\\ & + & (2q-2)\times 324+(p-2)(q-1)\times 400+(4q-4)\times 450+4(p-2)(q-1)\times 500\\ & + & 4\times 414+4(q-2)\times 432+2(p-2)\times 540+2(p-2)(q-2)\times 560\\ & + & 2(p-1)\times 575+(p-1)(q-2)\times 625+4\times 529+4\times 575+4(p-2)\times 621\\ & + & 4(q-2)\times 600+(4p-8)\times 675+4(p-2)(q-2)\times 700\\ & = & 6945pq-1740p-4167q+812. \end{array}$$

- **The *ve*-valency Randić descriptor**.

Using the statistics given in Table 4, we determine the *ve*-valency based Randić descriptor of *γ*(*p*, *q*) as:

$$\begin{array}{ccc} R^{ve}\left(\gamma \left(p,q\right)\right) & = & \sum_{uv\in E(\gamma (p,q)}{\left(d_{ve}\left(u\right)\times d_{ve}\left(v\right)\right)}^{-\frac{1}{2}},\\ R^{ve}\left(\gamma \left(p,q\right)\right) & = & 4\times 156^{-\frac{1}{2}}+4\times 168^{-\frac{1}{2}}+\left(2q-4\right)\times 169^{-\frac{1}{2}}+\left(4q-4\right)\times 234^{-\frac{1}{2}}+4\times 322\\ & + & \left(4p-8\right)\times 368^{-\frac{1}{2}}+4\times 276^{-\frac{1}{2}}+4\times 299^{-\frac{1}{2}}+\left(4q-8\right)\times 312^{-\frac{1}{2}}\\ & + & 4\times 322^{-\frac{1}{2}}+\left(2p-4\right)\times 432^{-\frac{1}{2}}+\left(2q-2\right)\times 324^{-\frac{1}{2}}+\left(p-2\right)\left(q-1\right)\times 400^{-\frac{1}{2}}\\ & + & \left(4q-4\right)\times 450^{-\frac{1}{2}}+4\left(p-2\right)\left(q-1\right)\times 500^{-\frac{1}{2}}+4\times 414^{-\frac{1}{2}}+4\left(q-2\right)\times 432^{-\frac{1}{2}}\\ & + & 2\left(p-2\right)\times 540^{-\frac{1}{2}}+2\left(p-2\right)\left(q-2\right)\times 560^{-\frac{1}{2}}+2\left(p-1\right)\times 575^{-\frac{1}{2}}\\ & + & \left(p-1\right)\left(q-2\right)\times 625^{-\frac{1}{2}}+4\times 529^{-\frac{1}{2}}+4\times 575^{-\frac{1}{2}}+4\left(p-2\right)\times 621^{-\frac{1}{2}}\\ & + & 4\left(q-2\right)\times 600^{-\frac{1}{2}}+\left(4p-8\right)\times 675^{-\frac{1}{2}}+4\left(p-2\right)\left(q-2\right)\times 700^{-\frac{1}{2}}\\ & = & (\frac{9}{100}+\frac{2\sqrt{5}}{25}+\frac{2\sqrt{7}}{35}+\frac{\sqrt{35}}{70})pq+(\frac{13\sqrt{3}}{90}+\frac{\sqrt{15}}{45}+\frac{7\sqrt{23}}{115}+\frac{4\sqrt{69}}{207}-\frac{\sqrt{35}}{35}\\ & - & \frac{2\sqrt{5}}{25}-\frac{4\sqrt{7}}{35}-\frac{13}{100})p+(\frac{\sqrt{78}}{39}+\frac{2\sqrt{2}}{15}+\frac{\sqrt{3}}{9}+\frac{\sqrt{6}}{15}+\frac{2\sqrt{26}}{39}-\frac{\sqrt{35}}{35}\\ & - & \frac{4\sqrt{5}}{25}-\frac{4\sqrt{7}}{35}+\frac{731}{5850})q-\frac{2\sqrt{2}}{15}-\frac{23\sqrt{3}}{45}+\frac{4\sqrt{5}}{25}+\frac{8\sqrt{7}}{35}+\frac{2\sqrt{35}}{35}-\frac{8731}{134550}\\ & - & \frac{2\sqrt{15}}{45}-\frac{2\sqrt{6}}{15}-\frac{8\sqrt{23}}{115}-\frac{2\sqrt{26}}{39}-\frac{2\sqrt{69}}{207}+\frac{4\sqrt{322}}{161}+\frac{4\sqrt{299}}{299}\\ & + & \frac{2\sqrt{39}}{39}+\frac{2\sqrt{46}}{69}+\frac{\sqrt{42}}{21}-\frac{2\sqrt{78}}{39}\\ & = & (0.50460)pq+(0.00838)p+(0.32806)q+0.03749. \end{array}$$

- **The *ev*-valency Randić descriptor**.

Using the statistics given in Table 2, we determine the *ev*-valency based Randić descriptor of *γ*(*p*, *q*) as:

$$\begin{array}{ccc} R^{ev}\left(\gamma \left(p,q\right)\right) & = & \sum_{e\in E(\gamma (p,q))}d_{ev}{\left(e\right)}^{-\frac{1}{2}},\\ R^{ev}\left(\gamma \left(p,q\right)\right) & = & \left(2q+4\right)\times 6^{-\frac{1}{2}}+\left(4q-4\right)\times 7^{-\frac{1}{2}}+\left(4p-4\right)\times 8^{-\frac{1}{2}}+\left(2p+4q\right)\times 9^{-\frac{1}{2}}\\ & + & p\left(q-1\right)\times 8^{-\frac{1}{2}}+4\left(p-1\right)\left(q-1\right)\times 9^{-\frac{1}{2}}+2p\left(q-1\right)\times 10^{-\frac{1}{2}}\\ & + & \left(p-1\right)q\times 10^{-\frac{1}{2}}+4\left(p-1\right)q\times 12^{-\frac{1}{2}}\\ & = & (\frac{\sqrt{2}}{4}+\frac{4}{3}+\frac{3\sqrt{10}}{10}+\frac{2\sqrt{3}}{3})pq+(\frac{3\sqrt{2}}{4}-\frac{2}{3}-\frac{\sqrt{10}}{5})p\\ & + & (\frac{\sqrt{6}}{3}+\frac{4\sqrt{7}}{7}-\frac{\sqrt{10}}{10}-\frac{2\sqrt{3}}{3})q+\frac{2\sqrt{6}}{3}-\frac{4\sqrt{7}}{7}-\sqrt{2}+\frac{4}{3}\\ & = & (3.7902)pq-(0.23853)p+(0.8575)q+0.0402 \end{array}$$

- **The atom-bond connectivity descriptor.**

Using the statistics given in Table 4, we determine the *ve*-valency based atom-bond connectivity descriptor of *γ*(*p*, *q*) as:

$$\begin{array}{ccc} {ABC}^{ve}\left(\gamma \left(p,q\right)\right) & = & \sum_{uv\in E(\gamma (p,q))}\sqrt{\frac{d_{ve}\left(u\right)+d_{ve}\left(v\right)-2}{d_{ve}\left(u\right)\times d_{ve}\left(v\right)}},\\ {ABC}^{ve}\left(\gamma \left(p,q\right)\right) & = & 4\times \sqrt{\frac{25-2}{156}}+4\times \sqrt{\frac{26-2}{168}}+\left(2q-4\right)\times \sqrt{\frac{26-2}{169}}\\ & + & \left(4q-4\right)\times \sqrt{\frac{31-2}{234}}+4\times \sqrt{\frac{37-2}{322}}+\left(4p-8\right)\times \sqrt{\frac{39-2}{368}}\\ & + & 4\times \sqrt{\frac{35-2}{276}}+4\times \sqrt{\frac{36-2}{299}}+\left(4q-8\right)\times \sqrt{\frac{37-2}{312}}\\ & + & 4\times \sqrt{\frac{37-2}{322}}+\left(2p-4\right)\times \sqrt{\frac{43-2}{432}}+\left(2q-2\right)\times \sqrt{\frac{36-2}{324}}\\ & + & \left(p-2\right)\left(q-1\right)\times \sqrt{\frac{40-2}{400}}+\left(4q-4\right)\times \sqrt{\frac{43-2}{450}}+4\left(p-2\right)\left(q-1\right)\times \sqrt{\frac{45-2}{500}}\\ & + & 4\times \sqrt{\frac{41-2}{414}}+4\left(q-2\right)\times \sqrt{\frac{42-2}{432}}+2\left(p-2\right)\times \sqrt{\frac{47-2}{540}}\\ & + & 2\left(p-2\right)\left(q-2\right)\times \sqrt{\frac{48-2}{560}}+2\left(p-1\right)\times \sqrt{\frac{48-2}{575}}+\left(p-1\right)\left(q-2\right)\times \sqrt{\frac{50-2}{625}}\\ & + & 4\times \sqrt{\frac{46-2}{529}}+4\times \sqrt{\frac{48-2}{575}}+4\left(p-2\right)\times \sqrt{\frac{50-2}{621}}\\ & + & 4\left(q-2\right)\times \sqrt{\frac{49-2}{600}}+\left(4p-8\right)\times \sqrt{\frac{52-2}{675}}+4\left(p-2\right)\left(q-2\right)\times \sqrt{\frac{53-2}{700}} \end{array}$$

$$\begin{array}{ccc} {ABC}^{ve}\left(\gamma \left(p,q\right)\right) & = & (\frac{\sqrt{1610}}{70}+\frac{2\sqrt{215}}{25}+\frac{2\sqrt{357}}{35}+\frac{\sqrt{38}}{20}+\frac{4\sqrt{3}}{25})pq+(\frac{\sqrt{3}}{75}-\frac{\sqrt{38}}{20}-\frac{4\sqrt{357}}{35}\\ & - & \frac{2\sqrt{215}}{25}-\frac{\sqrt{1610}}{35}+\frac{16\sqrt{23}}{69}+\frac{\sqrt{851}}{23}+\frac{4\sqrt{6}}{9}+\frac{\sqrt{123}}{18}+\frac{2\sqrt{2}}{5})p\\ & + & (\frac{4\sqrt{6}}{13}-\frac{4\sqrt{3}}{25}+\frac{2\sqrt{30}}{9}+\frac{\sqrt{2730}}{39}-\frac{4\sqrt{215}}{25}-\frac{\sqrt{38}}{10}+\frac{2\sqrt{82}}{15}+\frac{\sqrt{34}}{9}\\ & + & \frac{2\sqrt{754}}{39}+\frac{\sqrt{282}}{15}-\frac{4\sqrt{357}}{35}-\frac{\sqrt{1610}}{35})q+\frac{2\sqrt{2}}{5}-\frac{26\sqrt{3}}{75}-\frac{2\sqrt{2730}}{39}\\ & - & \frac{\sqrt{123}}{9}-\frac{2\sqrt{82}}{15}+\frac{4\sqrt{215}}{25}-\frac{2\sqrt{282}}{15}-\frac{2\sqrt{754}}{39}+\frac{2\sqrt{1794}}{69}+\frac{4\sqrt{230}}{23}\\ & + & \frac{4\sqrt{10166}}{299}+\frac{8\sqrt{357}}{35}-\frac{2\sqrt{851}}{23}-\frac{4\sqrt{30}}{9}+\frac{2\sqrt{897}}{39}-\frac{\sqrt{34}}{9}+\frac{4\sqrt{7}}{7}\\ & + & \frac{2\sqrt{1610}}{35}-\frac{176\sqrt{6}}{117}-\frac{32\sqrt{23}}{69}+\frac{\sqrt{38}}{10}+\frac{2\sqrt{253}}{23}+\frac{8\sqrt{11}}{23}\\ & = & (3.4112)pq-(0.11295)p+(1.1480)q+0.0437 \end{array}$$

- **The geometric-arithmetic descriptor**.

Using the statistics given in Table 4, we determine the *ve*-valency based geometric arithmetic descriptor of *γ*(*p*, *q*) as:

$$\begin{array}{ccc} {GA}^{ve}\left(\gamma \left(p,q\right)\right) & = & \sum_{uv\in E(\gamma (p,q))}\frac{2\sqrt{deg_{ve}\left(u\right)\times d_{ve}\left(v\right)}}{d_{ve}\left(u\right)+d_{ve}\left(v\right)},\\ {GA}^{ve}\left(\gamma \left(p,q\right)\right) & = & 4\times \frac{2\sqrt{156}}{25}+4\times \frac{2\sqrt{168}}{26}+\left(2q-4\right)\times \frac{2\sqrt{169}}{26}+\left(4q-4\right)\times \frac{2\sqrt{234}}{31}\\ & + & 4\times \frac{2\sqrt{322}}{37}+\left(4p-8\right)\times \frac{2\sqrt{368}}{39}+4\times \frac{2\sqrt{276}}{35}+4\times \frac{2\sqrt{299}}{36}\\ & + & \left(4q-8\right)\times \frac{2\sqrt{312}}{37}+4\times \frac{2\sqrt{322}}{37}+\left(2p-4\right)\times \frac{2\sqrt{432}}{43}+\left(2q-2\right)\times \frac{2\sqrt{324}}{36}\\ & + & \left(p-2\right)\left(q-1\right)\times \frac{2\sqrt{400}}{40}+\left(4q-4\right)\times \frac{2\sqrt{450}}{43}+4\left(p-2\right)\left(q-1\right)\times \frac{2\sqrt{500}}{45}\\ & + & 4\times \frac{2\sqrt{414}}{41}+4\left(q-2\right)\times \frac{2\sqrt{432}}{42}+2\left(p-2\right)\times \frac{2\sqrt{540}}{47}\\ & + & 2\left(p-2\right)\left(q-2\right)\times \frac{2\sqrt{560}}{48}+2\left(p-1\right)\times \frac{2\sqrt{575}}{48}+\left(p-1\right)\left(q-2\right)\times \frac{2\sqrt{625}}{50}\\ & + & 4\times \frac{2\sqrt{529}}{46}+4\times \frac{2\sqrt{575}}{48}+4\left(p-2\right)\times \frac{2\sqrt{621}}{50}\\ & + & 4\left(q-2\right)\times \frac{2\sqrt{600}}{49}+\left(4p-8\right)\times \frac{2\sqrt{675}}{52}+4\left(p-2\right)\left(q-2\right)\times \frac{2\sqrt{700}}{53}\\ & = & (\frac{\sqrt{35}}{3}+2+\frac{80\sqrt{7}}{53}+\frac{16\sqrt{5}}{9})pq+(-\frac{2\sqrt{35}}{3}+\frac{24\sqrt{15}}{47}+\frac{12\sqrt{69}}{25}-\frac{160\sqrt{7}}{53}\\ & - & \frac{16\sqrt{5}}{9}+\frac{1914\sqrt{3}}{559}+\frac{193\sqrt{23}}{156}-3)p+(-\frac{2\sqrt{35}}{3}+\frac{16\sqrt{78}}{37}+\frac{80\sqrt{6}}{49}+\frac{24\sqrt{26}}{31}\\ & - & \frac{160\sqrt{7}}{53}-\frac{32\sqrt{5}}{9}+\frac{120\sqrt{2}}{43}+\frac{16\sqrt{3}}{7}+1)q+2+\frac{4\sqrt{35}}{3}-\frac{32\sqrt{78}}{37}\\ & - & \frac{48\sqrt{15}}{47}-\frac{160\sqrt{6}}{49}-\frac{24\sqrt{26}}{31}-\frac{88\sqrt{69}}{175}+\frac{8\sqrt{42}}{13}-\frac{191\sqrt{23}}{156}+\frac{2\sqrt{299}}{9}\\ & + & \frac{320\sqrt{7}}{53}+\frac{32\sqrt{5}}{9}-\frac{44684\sqrt{3}}{3913}-\frac{120\sqrt{2}}{43}+\frac{16\sqrt{322}}{37}+\frac{24\sqrt{46}}{41}+\frac{16\sqrt{39}}{25}\\ & = & (11.941)pq-(1.0778)p+(0.7895)q+0.0565 \end{array}$$

- **The harmonic descriptor.**

Using the statistics given in Table 4, we determine the *ve*-valency based Harmonic descriptor of *γ*(*p*, *q*) as:

$$\begin{array}{ccc} H^{ve}\left(\gamma \left(p,q\right)\right) & = & \sum_{uv\in E(\gamma (p,q))}\frac{2}{d_{ve}\left(u\right)+d_{ve}\left(v\right)},\\ H^{ve}\gamma \left(p,q\right)) & = & 4\times \frac{2}{25}+4\times \frac{2}{26}+\left(2q-4\right)\times \frac{2}{26}+\left(4q-4\right)\times \frac{2}{31}+4\times \frac{2}{37}\\ & + & \left(4p-8\right)\times \frac{2}{39}+4\times \frac{2}{35}+4\times \frac{2}{36}+\left(4q-8\right)\times \frac{2}{37}+4\times \frac{2}{37}\\ & + & \left(2p-4\right)\times \frac{2}{43}+\left(2q-2\right)\times \frac{2}{36}+\left(p-2\right)\left(q-1\right)\times \frac{2}{40}\\ & + & \left(4q-4\right)\times \frac{2}{43}+4\left(p-2\right)\left(q-1\right)\times \frac{2}{45}+4\times \frac{2}{41}+4\left(q-2\right)\times \frac{2}{42}\\ & + & 2\left(p-2\right)\times \frac{2}{47}+2\left(p-2\right)\left(q-2\right)\times \frac{2}{48}+2\left(p-1\right)\times \frac{2}{48}\\ & + & \left(p-1\right)\left(q-2\right)\times \frac{2}{50}+4\times \frac{2}{46}+4\times \frac{2}{48}+4\left(p-2\right)\times \frac{2}{50}\\ & + & 4\left(q-2\right)\times \frac{2}{49}+\left(4p-8\right)\times \frac{2}{52}+4\left(p-2\right)\left(q-2\right)\times \frac{2}{53}\\ & = & \frac{5987}{11925}pq+\frac{1286462}{313305525}p+\frac{117984725506}{374653413225}q+\frac{23283715095307}{598378880271900}\\ & = & (0.50205)pq+(0.0041061)p+(0.31492)q+0.038911 \end{array}$$

- **The sum-connectivity descriptor.**

Using the statistics given in Table 4, we determine the *ve*-valency based sum connectivity descriptor of *γ*(*p*, *q*) as:

$$\begin{array}{ccc} \chi^{ve}\left(\gamma \left(p,q\right)\right) & = & \sum_{uv\in E(\gamma (p,q))}{\left(d_{ve}\left(u\right)+d_{ve}\left(v\right)\right)}^{-\frac{1}{2}},\\ \chi^{ve}\left(\gamma \left(p,q\right)\right) & = & 4\times {\left(25\right)}^{-\frac{1}{2}}+4\times {\left(26\right)}^{-\frac{1}{2}}+\left(2q-4\right)\times {\left(26\right)}^{-\frac{1}{2}}+\left(4q-4\right)\times {\left(31\right)}^{-\frac{1}{2}}\\ & + & 4\times {\left(37\right)}^{-\frac{1}{2}}+\left(4p-8\right)\times {\left(39\right)}^{-\frac{1}{2}}+4\times {\left(35\right)}^{-\frac{1}{2}}+4\times {\left(36\right)}^{-\frac{1}{2}}+\left(4q-8\right)\times {\left(37\right)}^{-\frac{1}{2}}\\ & + & 4\times {\left(37\right)}^{-\frac{1}{2}}+\left(2p-4\right)\times {\left(43\right)}^{-\frac{1}{2}}+\left(2q-2\right)\times {\left(36\right)}^{-\frac{1}{2}}+\left(p-2\right)\left(q-1\right)\times {\left(40\right)}^{-\frac{1}{2}}\\ & + & \left(4q-4\right)\times {\left(43\right)}^{-\frac{1}{2}}+4\left(p-2\right)\left(q-1\right)\times {\left(45\right)}^{-\frac{1}{2}}+4\times {\left(41\right)}^{-\frac{1}{2}}+4\left(q-2\right)\times {\left(42\right)}^{-\frac{1}{2}}\\ & + & 2\left(p-2\right)\times {\left(47\right)}^{-\frac{1}{2}}+2\left(p-2\right)\left(q-2\right)\times {\left(48\right)}^{-\frac{1}{2}}+2\left(p-1\right)\times {\left(48\right)}^{-\frac{1}{2}}\\ & + & \left(p-1\right)\left(q-2\right)\times {\left(50\right)}^{-\frac{1}{2}}+4\times {\left(46\right)}^{-\frac{1}{2}}+4\times {\left(48\right)}^{-\frac{1}{2}}+4\left(p-2\right)\times {\left(50\right)}^{-\frac{1}{2}}\\ \; & + & 4\left(q-2\right)\times {\left(49\right)}^{-\frac{1}{2}}+\left(4p-8\right)\times {\left(52\right)}^{-\frac{1}{2}}+4\left(p-2\right)\left(q-2\right)\times {\left(53\right)}^{-\frac{1}{2}}\\ & = & (\frac{\sqrt{10}}{20}+\frac{4\sqrt{53}}{53}+\frac{\sqrt{2}}{10}+\frac{\sqrt{3}}{6}+\frac{4\sqrt{5}}{15})pq+(-\frac{\sqrt{10}}{20}+\frac{2\sqrt{43}}{43}+\frac{4\sqrt{39}}{39}\\ & - & \frac{8\sqrt{53}}{53}+\frac{2\sqrt{13}}{13}+\frac{\sqrt{2}}{5}-\frac{\sqrt{3}}{6}+\frac{2\sqrt{47}}{47}-\frac{4\sqrt{5}}{15})p+(\frac{\sqrt{26}}{13}+\frac{4\sqrt{37}}{37}+\frac{4\sqrt{43}}{43}\\ & + & \frac{4\sqrt{31}}{31}-\frac{8\sqrt{53}}{53}-\frac{8\sqrt{5}}{15}-\frac{\sqrt{3}}{3}-\frac{\sqrt{2}}{10}+\frac{2\sqrt{42}}{21}+\frac{19}{21}-\frac{\sqrt{10}}{10})q-\frac{3\sqrt{2}}{5}+\frac{5\sqrt{3}}{6}\\ & + & \frac{8\sqrt{5}}{15}+\frac{\sqrt{10}}{10}+\frac{4\sqrt{35}}{35}-\frac{4\sqrt{13}}{13}-\frac{8\sqrt{39}}{39}+\frac{2\sqrt{46}}{23}-\frac{4\sqrt{42}}{21}-\frac{1}{105}-\frac{4\sqrt{47}}{47}\\ & - & \frac{8\sqrt{43}}{43}+\frac{4\sqrt{41}}{41}+\frac{16\sqrt{53}}{53}-\frac{4\sqrt{31}}{31}\\ & = & (1.7340)pq-(0.06723)p+(0.57366)q+(0.03589). \end{array}$$

## 5 Numerical and graphical representation and discussion

**Fig 2:**
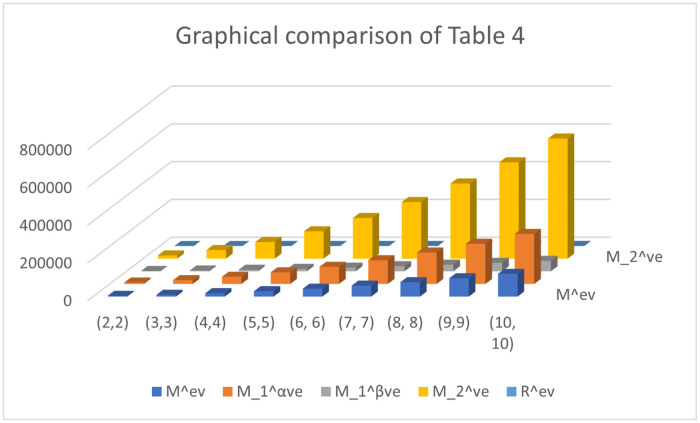
Graphical Comparison of $M^{ev},M_{1}^{\alpha ve}M_{1}^{\beta ve},$, $M_{2}^{ve}$ and *R*^*ev*^.

**Fig 3:**
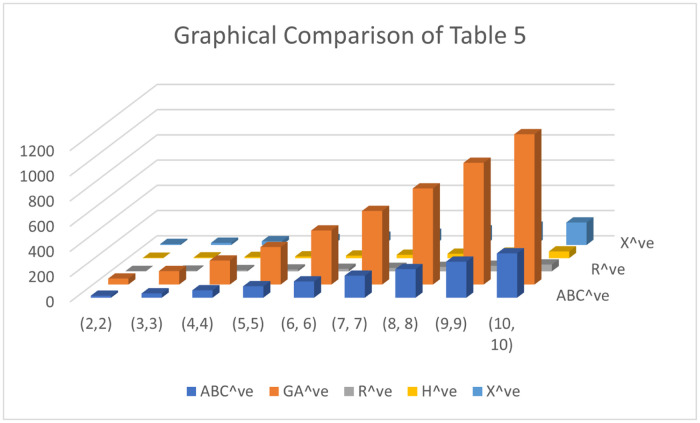
Graphical Comparison of *ABC*^*ve*^, *GA*^*ve*^, *R*^*ve*^, *H*^*ve*^ and *χ*^*ve*^.

Both numerical and graphical calculations are used to determine the *ve* and *ev* for ten distinct categories of valency-based topological descriptors for the *γ*(*p*, *q*). The behavior of the first Zagreb alpha descriptor, first Zagreb beta descriptor, and second Zagreb descriptor is practically identical in the growing orientation as the quantity of *n* rises, while the appreciate of the *ev* Zagreb descriptor improves very quickly with the increased value of *n*, as can be seen in Fig 2. The patterns of behavior of the atom bond connectivity descriptor and geometric arithmetic descriptor are hardly noticeably growing with an elevated value of *n*, but the value of the *ev* Randić descriptor has a very quick rise with the increased value of *n*, as can be seen in Fig 3.

The numerical and graphical representations of *γ*(*p*, *q*) are shown below.

## 6 Closing remarks

Topological indicators have several uses in fields such as chemical graph theory, computer science, networks, agriculture, etc. These characteristics aid in determining how their frameworks behave. We generated 10 distinct sorts of topological descriptors based on *ev* and *ve* valency for the *γ*-sheet of boron clusters. Their numerical values for different amounts of *n* have been computed after their clear formulations have been determined. In addition, we compared the charts and spoke about their behavior. We note that when the quantity of *n* grows, all descriptor values rise, results are shown in the Tables 5 and 6.

**Table 5:** Numerical comparison of $M^{ev},M_{1}^{\alpha ve},M_{1}^{\beta ve}$, $M_{2}^{ve}$ and *R*^*ev*^.

(*p*, *q*)	*M* ^*ev*^	$M_{1}^{\alpha ve}$	$M_{1}^{\beta ve}$	$M_{2}^{ve}$	*R* ^*ev*^
(2,2)	3916	7756	1788	16778	16.43894
(3, 3)	9658	20042	4408	45596	36.00891
(4,4)	17928	37996	8184	88304	63.15928
(5,5)	28726	61618	13116	144902	97.89005
(6, 6)	42052	90908	19204	215390	140.20122
(7, 7)	57906	125866	26448	299768	190.09279
(8, 8)	76288	166492	34848	398036	247.56476
(9,9)	97198	212786	44404	510194	312.61713
(10, 10)	120636	264748	55116	636242	385.24990

**Table 6:** Numerical comparison of *ABC*^*ve*^, *GA*^*ve*^, *R*^*ve*^, *H*^*ve*^ and *χ*^*ve*^.

(*p*, *q*)	*ABC* ^*ve*^	*GA* ^*ve*^	*R* ^*ve*^	*H* ^*ve*^	*χ* ^*ve*^
(2,2)	15.75860	47.2439	2.72877	2.6851632	7.98475
(3, 3)	33.84965	106.6606	5.58821	5.5144393	17.16118
(4,4)	58.76310	189.9593	9.45685	9.3478154	29.80561
(5,5)	90.49895	297.1400	14.33469	14.1852915	45.91804
(6, 6)	129.05720	428.2027	20.22173	20.0268676	65.49847
(7, 7)	174.43785	583.1474	27.11797	26.8725437	88.54690
(8, 8)	226.64090	761.9741	35.02341	34.7223198	115.06333
(9,9)	285.66635	964.6828	43.93805	43.5761959	145.04776
(10, 10)	351.51420	1191.2735	53.86189	53.4341720	178.50019

### 6.1 Future directions and open problems

Researchers working in topological numbers of graphs will find guidance and direction in this section. There are many topological numbers, like connection number based topological index is one the next work can be done after this chosen topic.

### 6.2 Limitations of the proposed model

There are few limitations of this work, like *Computational Complexity:* Certain topological indices or computational models may exhibit significant computational complexity, rendering them unfeasible for handling extensive datasets or real-time applications.
